# Chlorpyrifos Oxon Activates Glutamate and Lysine for Protein Cross-linking

**DOI:** 10.1021/acs.chemrestox.2c00333

**Published:** 2023-01-04

**Authors:** Diego Muñoz-Torrero, Lawrence M. Schopfer, Oksana Lockridge

**Affiliations:** † Laboratory of Medicinal Chemistry (CSIC Associated Unit), Faculty of Pharmacy and Food Sciences, and Institute of Biomedicine (IBUB), University of Barcelona, Barcelona 08028, Spain; ‡ University of Nebraska Medical Center, Omaha, Nebraska 68198, United States

## Abstract

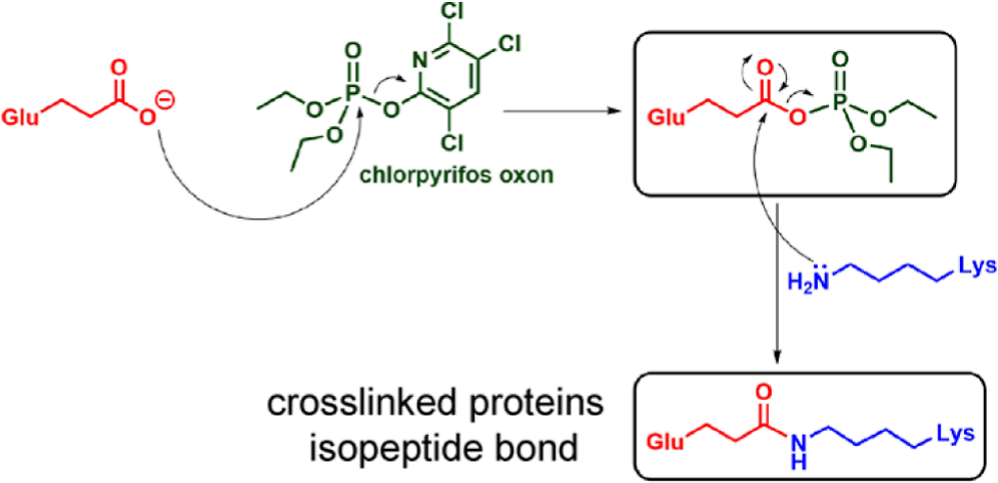

Chronic low-dose exposure to organophosphorus (OP) toxicants is correlated with an increase in the risk of impaired cognition and neurodegenerative diseases. A mechanism to explain this relationship is needed. We suggest that the formation of organophosphate-induced high-molecular-weight protein aggregates that disrupt cell function may be the missing link. It has been demonstrated that such aggregation can be promoted by OP-labeled lysine. Alternatively, OP-labeled glutamate may be the initiator. To test this hypothesis, we treated MAP-rich tubulin *Sus scrofa* and human transglutaminase with chlorpyrifos oxon. Trypsin-digested proteins were subjected to liquid chromatography–tandem mass spectrometry followed by Protein Prospector searches to identify diethyl phosphate adducts and cross-linked peptides. We report the presence of diethyl phosphate adducts on the side chains of glutamate, lysine, and tyrosine, as well as cross-links between glutamate and lysine. Glutamate-lysine cross-linking could be initiated either by diethyl phosphate-activated glutamate or by diethyl phosphate-activated lysine to form stable isopeptide bonds between and within proteins. It was concluded that organophosphate-induced high-molecular-weight protein aggregates could promote brain dysfunction.

## Introduction

Twenty years ago, epidemiological studies indicated that chronic, low-dose exposure to organophosphates was related to neurological deficits.^1−4^ Low-dose exposure is defined as exposure to levels of organophosphate that do not result in cholinergic symptoms typical of inhibition of acetylcholinesterase. These studies suggested that the neurological defects were the consequence of organophosphorus (OP) compounds reacting with proteins other than cholinesterase.

In 2005, we initiated studies to identify noncholinesterase proteins that covalently bind low doses of OP compounds.^5^ We found that albumin, carboxylesterase, and eight other unidentified proteins in mouse blood were labeled. Subsequently, we showed that bovine albumin was labeled on tyrosine-410.^6^ As it turns out, in 1962, Sanger showed that the equivalent position in human albumin, tyrosine-411, was labeled by diisopropylphosphate.^7^ Additional work has shown that proteins form covalent adducts on tyrosine with a variety of OP compounds.^8−15^ We also found that OP makes covalent bonds with the side chain of lysine.^11,14,16,17^

Some of the lysine adducts proceed to form high-molecular-weight cross-linked proteins.^17−19^ The OP-induced cross-link is a zero-length isopeptide bond between the ε-amino group of lysine and the γ-carboxyl group of glutamic acid. During a presentation of these results at the 14th International Meeting on Cholinesterases/8th International Conference on Paraoxonases in Bologna, Italy (Sept 2022), Diego Muñoz-Torrero, a medicinal chemist, suggested protein cross-linking could also be initiated by an OP adduct on glutamate.

The current study found evidence for OP-labeled glutamate residues in proteins that have been exposed to chlorpyrifos oxon (CPO). We chose CPO because it readily makes adducts on proteins. In addition, we tested the possibility that other nucleophilic amino acids (arginine, histidine, threonine, aspartate, and serine) might also form CPO adducts. Of these, we found mass spectral evidence of OP adducts on glutamate, serine, aspartate, and threonine. We proposed mechanisms for creating isopeptide cross-links between diethylphosphoglutamate and lysine. Finally, we explored the feasibility of forming isopeptide cross-links between diethylphosphotyrosine and lysine.

## Materials and Methods

Orbitrap mass spectrometry (MS) raw files deposited at the ProteomeXchange Consortium^20,21^ with identifier PXD034529^19^ were searched for diethyl phosphate adducts on Glu, Asp, Lys, Tyr, His, Arg, Ser, and Thr and for isopeptide cross-linked peptides. We used Protein Prospector software to search for peptides whose monoisotopic mass was increased 136.03 Da by covalent binding of diethyl phosphate. This added mass is the monoisotopic mass of diethyl phosphate minus a proton lost from the nucleophile upon reaction, resulting in a molecular composition of C_4_H_9_O_3_P.

Microtubule-associated protein-rich tubulin (MAP-rich tubulin) proteins from porcine brains (Cytoskeleton Inc. #ML116) were incubated with 200 μM CPO in 20 mM Tris–HCl pH 8.5, with 0.01% azide at 37 °C for 2 days. Excess CPO was removed by dialysis before proteins were separated by SDS gel electrophoresis. Proteins in Coomassie blue-stained gel slices were reduced with dithiothreitol, alkylated with iodoacetamide, and digested with trypsin.^19^ Tryptic peptides were subjected to liquid chromatography–tandem mass spectrometry (MS/MS) on an Orbitrap Fusion Lumos Tribrid mass spectrometer.^19^

Recombinant human transglutaminase (Zedira GmbH T002) in 20 mM imidazole pH 7.5, with 0.15 M NaCl was treated with 100 μM CPO (Chem Service Inc. MET-11459B). After excess CPO was removed, the protein was reduced with dithiothreitol, alkylated with iodoacetamide, digested with trypsin, and subjected to liquid chromatography–MS/MS on an Orbitrap Fusion Lumos Tribrid mass spectrometer. Trypsin digested proteins were searched using Protein Prospector software.

The reaction of CPO with the side chains of glutamic acid or lysine adds 136.03 Da to the amino acid. Protein Prospector software was used to search for an added mass of 136.03 Da on peptides. An MS/MS spectrum was accepted as evidence of an OP adduct only if fragment ions defined the exact location of the adduct. Signature ions for OP-lysine and OP-tyrosine were often present in MS/MS spectra. Signature ions for adducts on lysine have masses 237 and 220 Da, with 237 observed most frequently. Signature ions for adducts on tyrosine have masses 272, 244, 226, 216, and 198 Da, with 272 observed most frequently. Signature ions for adducts on glutamate were calculated at 238, 210, 192, 182, and 164 Da, though these ions for Dep-Glu were not observed. The presence of signature ions confirmed the interpretation of adducts with an added mass of 136.03 Da.

Protein Prospector software was also used to search for cross-linked peptides. The bridge element composition for peptides cross-linked with the loss of a water molecule was H-2 O-1. An MS/MS spectrum was accepted as evidence of a cross-linked peptide if it met the following criteria. (1) More than 40% of the peaks in the MS/MS spectrum are assigned to the cross-linked peptides. (2) At least 2 cross-link specific ions are present in a series that defines an amino acid. Cross-link specific ions are defined as fragment ions that contain sequences from both peptides. (3) Fragment ions from both ions must be present. Generally, one peptide is well defined by fragment ions, but the second peptide is not. The less well-defined peptide must show fragment ions in a series consistent with the sequence of the second peptide. (4) Manual evaluation of the MS/MS spectrum agrees with the software-assigned cross-link. When manual evaluation found that the cross-link specific ions fit an unrelated peptide sequence, the candidate cross-link was judged to be a false positive. (5) For the specific case of peptides cross-linked by the mechanisms in Figure 3, we checked the parent ion mass using the Proteomics Toolkit.

A 4–20% polyacrylamide gradient gel was poured in a Hoefer apparatus. MAP-rich tubulin treated with CPO was reduced with dithiothreitol in SDS gel loading buffer in preparation for electrophoresis.

## Results

### Protein Aggregation

**Figure 1:**
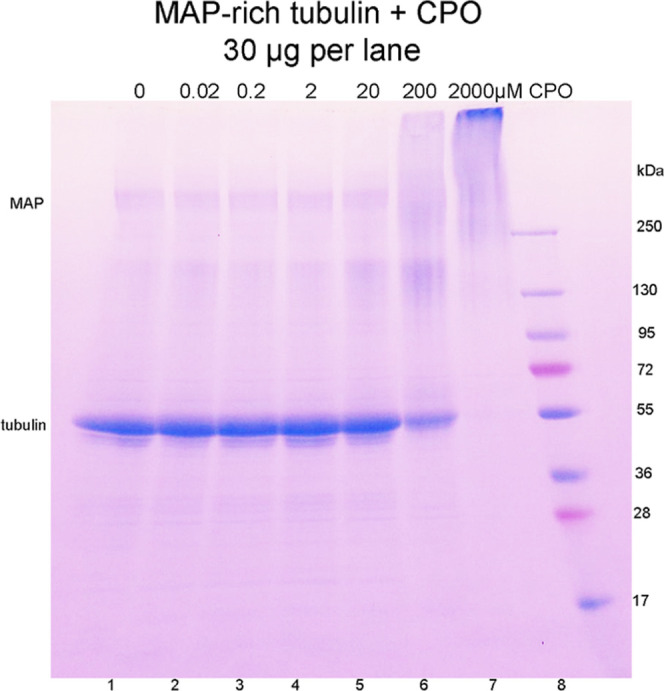
Protein cross-linking effect of CPO is visualized on an SDS gel stained with Coomassie blue. MAP-rich tubulin in 0.1 mL of 20 mM Tris–HCl pH 8.5, with 0.01% sodium azide was incubated with 0–2000 μM CPO at room temperature in a humidified chamber for 11 days. The control sample (lane 1) was treated with 2 μl acetonitrile. Dithiothreitol-reduced proteins were loaded at 30 μg per lane.

The cross-linking effect of CPO on MAP-rich tubulin *Sus scrofa* is visualized on the SDS gel in Figure 1. At 30 μg protein per lane, CPO effects were seen at concentrations as low as 0.02 μM (lane 2) visualized as a broad area at ∼200 kDa. This area is prominent in the 20 and 200 μM CPO-treated samples (lanes 5 and 6). The 200 μM CPO-treated sample (lane 6) has lost intensity in the tubulin band at 55 kDa and acquired aggregated protein bands of high molecular weight exceeding the 250 kDa marker. The 2000 μM CPO-treated sample (lane 7) has almost no tubulin at 55 kDa and the protein becomes cross-linked to high-molecular-weight aggregates.

### CPO Adducts

MAP-rich tubulin proteins, treated with 200 μM CPO, were digested with trypsin and subjected to liquid chromatography–MS/MS. MS files were searched for diethyl phosphate adducts (Dep) using Protein Prospector. Table 1 shows sequences of peptides with an added mass of 136.03 Da for diethyl phosphate. We found 5 peptides covalently modified on glutamate (E), 6 modified on lysine (K), 17 modified on tyrosine (Y), and 1 peptide modified on serine (S). We found no diethyl phosphate adducts on aspartate (D), histidine (H), arginine (R), and threonine (T) and none in untreated control proteins.

**Table 1:** CPO Adducts (+136.03 Da) on MAP-rich Tubulin *S. scrofa*^a^

adduct	protein name	amino acid sequence	accession number
Dep-E	tubulin α 1A	APVISA**E**_**279**_KAY	NP_001302639
Dep-E	tubulin α 1A	AFVHWYVG**E**_**411**_GMEEGEFSEAR	NP_001302639
Dep-E	tubulin β 4B	**E**_**111**_LVDSVLDVVR	XP_003122400
Dep-E	MAP2 X8	LPLDVMKNEIVA**E**_**418**_ASPFA	XP_013839898
Dep-E	MAP2 X8	MT**E**_**1494**_QLETIPK	XP_013839898
Dep-K	tubulin α 1A	FDLMYA**K**_**401**_R	NP_001302639
Dep-K	tubulin β 4B	MSM**K**_**324**_EVDEQMLNVQNK	XP_003122400
Dep-K	tubulin β 4B	**K**_**352**_LAVNMVPFPR	XP_003122400
Dep-K	tubulin β 4B	STAIQELF**K**_**379**_R	XP_003122400
Dep-K	tubulin β 4B	**K**_**392**_AFLH	XP_003122400
Dep-K	MAP2 X8	**K**_**1576**_FILKPAIK	XP_013839898
Dep-Y	tubulin α 1A	GH**Y**_**108**_TIGK	NP_001302639
Dep-Y	tubulin α 1A	**Y**_**108**_TIGKEIIDL	NP_001302639
Dep-Y	tubulin α 1A	LSVD**Y**_**161**_GK	NP_001302639
Dep-Y	tubulin α 1A	PT**Y**_**224**_TNLNR	NP_001302639
Dep-Y	tubulin α 1A	NLDIERPT**Y**_**224**_TNLNR	NP_001302639
Dep-Y	tubulin α 1A	LVP**Y**_**262**_PR	NP_001302639
Dep-Y	tubulin α 1A	IHFPLAT**Y**_**272**_APVISAEK	NP_001302639
Dep-Y	tubulin α 1A	LAT**Y**_**272**_APVISAEK	NP_001302639
Dep-Y	tubulin α 1A	A**Y**_**282**_HEQLSVAEITNACFEPANQMVKC	NP_001302639
Dep-Y	tubulin α 1A	VGIN**Y**_**357**_QPPTVVPGGDLAK	NP_001302639
Dep-Y	tubulin α 1A	LDHKFDLM**Y**_**399**_AK	NP_001302639
Dep-Y	tubulin β 4B	GH**Y**_**106**_TEGAELVDSVLDVVR	XP_003122400
Dep-Y	tubulin β 4B	EE**Y**_**159**_PDR	XP_003122400
Dep-Y	tubulin β 4B	IREE**Y**_**159**_PDR	XP_003122400
Dep-Y	tubulin β 4B	SS**Y**_**340**_FVEWIPNNVK	XP_003122400
Dep-Y	tubulin β 4B	LHW**Y**_**398**_TGEGMDEM	XP_003122400
Dep-Y	MAP2 X8	TPGTPGTPS**Y**_**1771**_PR	XP_013839898
Dep-S	tubulin α 1A	**S**_**140**_FGGGTGSGFTSLLMER	NP_001302639

a MAP-rich tubulin *S. scrofa* was incubated with 200 μM CPO. Dep-E = diethylphospho-glutamate; Dep-K = diethylphospho-lysine; Dep-Y = diethylphospho-tyrosine; Dep-S = diethylphospho-serine. The position of the labeled amino acid in the protein sequence is indicated. Protein accession numbers are from the National Center for Biotechnology Information database.

**Figure 2:**
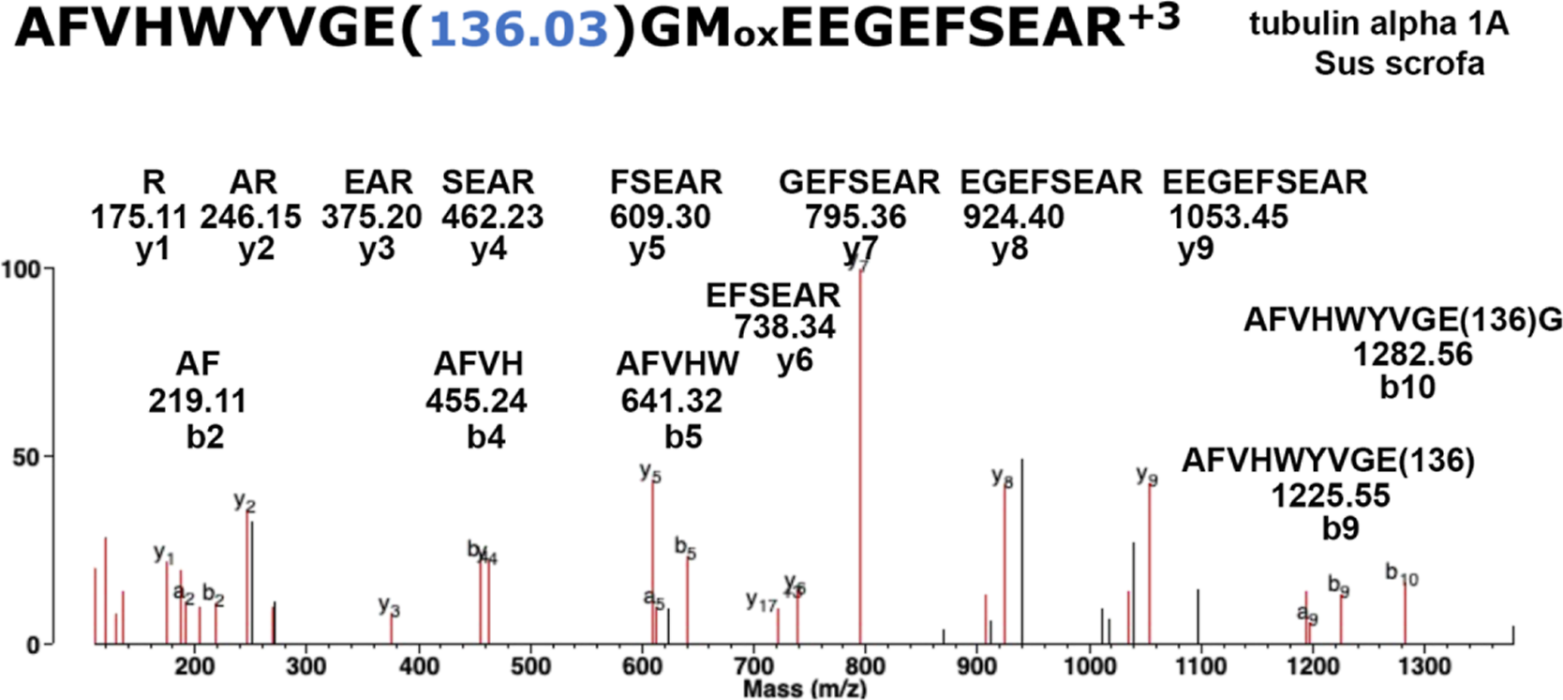
MS/MS spectrum showing the diethyl phosphate adduct on glutamic acid (E) in tubulin α 1A *S. scrofa* (accession NP_001302639). The peptide is identified by the y1 to y9 ion series and the b2 to b10 ion series whose structures are shown. The exact location of the 136.03 Da adduct on E is defined by the b9 ion at *m*/*z* 1225.55 and the b10 ion at *m*/*z* 1282.56. Oxidized methionine is indicated as Mox. The MH^3+^ parent ion has a mass of *m*/*z* 827.0506.

MS evidence for a diethyl phosphate adduct on glutamate is shown in Figure 2. The b ion fragments at *m*/*z* 1282.56 and 1225.55 convincingly identify the indicated E (residue 9 from the N-terminus) as the modified residue. Four other E residues in the peptide are ruled out as modified residues based on the masses of the y1 to y9 ion series that do not include the added mass of 136 Da. Representative MS/MS spectra for diethyl phosphate peptide adducts on lysine and tyrosine are in Figures S1 and S2 in the Supporting Information section.

Human transglutaminase was treated with 100 μM CPO, digested with trypsin, and subjected to liquid chromatography–MS/MS. MS files were searched for diethyl phosphate adducts (Dep) using Protein Prospector. Table 2 shows sequences of transglutaminase peptides with an added mass of 136.03 Da. 1 peptide carries the adduct on aspartate (D), 2 on glutamate (E), 8 on lysine (K), 1 on threonine (T), and 13 on tyrosine (Y). There were no diethyl phosphate adducts on histidine (H), serine (S), or arginine (R), and none on untreated control protein.

**Table 2:** CPO Adducts (+136.03) on Human Transglutaminase (TG2)^a^

adduct	protein name	amino acid sequence	accession number
Dep-D	TG2	**D**_**232**_**D**_**233**_QGVLLGR	P21980
Dep-E	TG2	CDL**E**_**13**_LETNGR	P21980
Dep-E	TG2	KYRDCLT**E**_**557**_SNLIK	P21980
Dep-K	TG2	E**K**_**30**_LVVR	P21980
Dep-K	TG2	IYQGSA**K**_**173**_FIK	P21980
Dep-K	TG2	QEYVLTQQGFIYQGSA**K**_**173**_FIK	P21980
Dep-K	TG2	SLIVGL**K**_**425**_ISTK	P21980
Dep-K	TG2	SVPLCILYE**K**_**550**_YR	P21980
Dep-K	TG2	ILGEP**K**_**598**_QK	P21980
Dep-K	TG2	**K**_**663**_LVVNFESDK	P21980
Dep-K	TG2	AV**K**_**677**_GFR	P21980
Dep-T	TG2	QEYVL**T**_**162**_QQGFIYQGSAK	P21980
Dep-Y	TG2	N**Y**_**50**_EASVDSLTF	P21980
Dep-Y	TG2	AWCPADAV**Y**_**149**_LDSEEER	P21980
Dep-Y	TG2	QE**Y**_**15**9_VLTQQGF	P21980
Dep-Y	TG2	QE**Y**_**159**_VLTQQGFIYQGSAK	P21980
Dep-Y	TG2	NSAHDQNSNLLIE**Y**_**315**_FR	P21980
Dep-Y	TG2	VVTNYNSAHDQNSNLLIE**Y**_**315**_FR	P21980
Dep-Y	TG2	TRPDLQPG**Y**_**351**_EGWQALDPTPQEK	P21980
Dep-Y	TG2	**Y**_**388**_DAPFVFAEVNADVVDWIQQDDGSVHK	P21980
Dep-Y	TG2	EDITHT**Y**_**443**_K	P21980
Dep-Y	TG2	**Y**_**445**_PEGSSEER	P21980
Dep-Y	TG2	ITNNTAEE**Y**_**503**_VCR	P21980
Dep-Y	TG2	**Y**_**528**_LLNLNLEPFSEK	P21980
Dep-Y	TG2	ALLVEPVINS**Y**_**575**_LLAER	P21980

a Human TG2 was incubated with 100 μM CPO. Dep-D = diethylphospho-aspartate; Dep-E = diethylphospho-glutamate; Dep-K = diethylphospho-lysine; Dep-T = diethylphospho-threonine; Dep-Y = diethylphospho-tyrosine. The position of the labeled amino acid in the protein sequence is indicated. The accession number is from the UniProt database.

**Figure 3:**
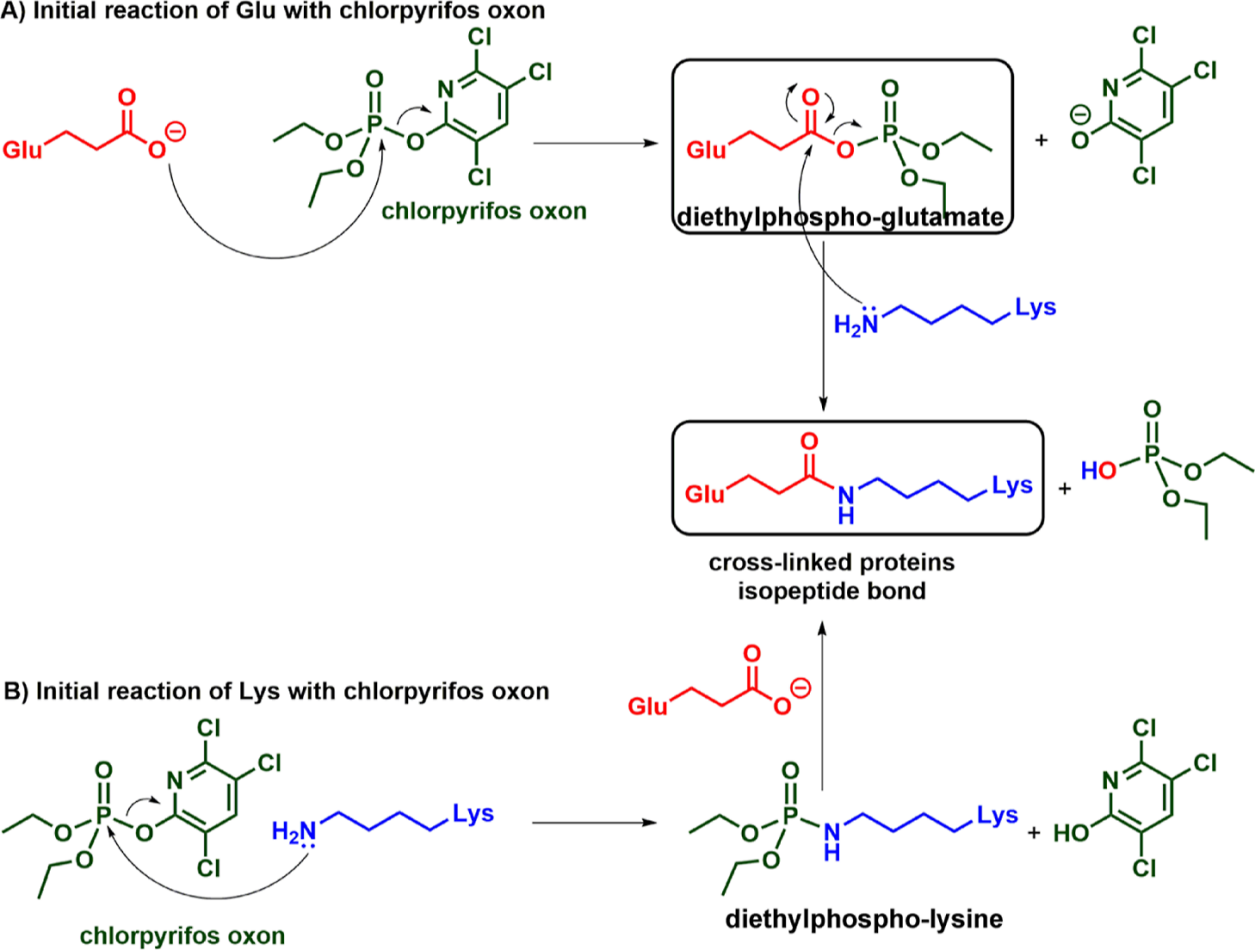
Proposed mechanisms of OP-induced protein cross-linking. In (A), the reaction is initiated by activated glutamate. In (B), the reaction is initiated by activated lysine. Both pathways link proteins through an isopeptide bond. Activated glutamate and activated lysine are defined as residues modified by diethyl phosphate. The mass of peptides cross-linked *via* an isopeptide bond is lower by 18 Da compared to the mass of each individual peptide. This is due to the loss of OH from Glu and H from Lys. Although Glu is negatively charged in solution, at neutral pH, as illustrated, it is positively charged when introduced into the mass spectrometer in 0.1% formic acid. Therefore, the observed mass change upon isopeptide bond formation is −18 Da for the loss of OH from Glu and H from Lys.

Diethylphospho-glutamate (Dep-E) adducts were found in four different proteins. This supports the idea that the cross-linking mechanism may be initiated by activated glutamate. Cross-linking initiated by either diethylphospho-E or diethylphospho-K yields the identical isopeptide bond (Figure 3). Fragmentation spectra of cross-linked peptides make no distinction between isopeptide bonds created by reaction of diethylphospho-glutamate with lysine or diethylphospho-lysine with glutamate.

### Identification of CPO-Induced Protein Cross-links Containing an Isopeptide Bond

The MS files that yielded information on diethyl phosphate adducts in Tables 1 and 2 also contained information on cross-linked proteins. One cross-linked peptide pair was identified in the MAP-rich tubulin data and one in the transglutaminase data. Table 3 indicates an isopeptide cross-link between tubulin α 1A and MAP2 X8. Table 3 also shows an internal cross-link in human transglutaminase between E523 and K527, where three residues lie between E and K in the sequence ECGTK.

**Table 3:** CPO-Induced Protein Cross-links Where the Zero-Length Isopeptide Bond Is between Glutamate and Lysine

Xlink	sequence	X-linked proteins	accession numbers
KE	TIGGGDDSFNTFFS**E**_**55**_TGA	tubulin α 1A	NP_001302639
	ST**K**_**1635**_SPR	MAP2 X8	XP_013839898
KE	GT**K**_**527**_YLLNLNLEPFSEK	human TG2	P21980
	TVSYNGILGP**E**_**523**_CG	human TG2	P21980

The cross-linked peptides in Table 3 could originate from a reaction with either activated glutamate (E) or activated lysine (K). Figure 3 shows mechanisms for both possibilities. OP binding to the side chains of glutamate or lysine activates these residues for cross-linking.

The mixed carboxylic–phosphoric anhydride formed by reaction of glutamate with CPO would be expected to be quite reactive toward nucleophiles, such as Lys-NH_2_. Carboxylic acid derivatives react with nucleophiles through an overall nucleophilic substitution reaction that proceeds in two steps, an initial nucleophilic addition to the electrophilic carbonyl carbon atom and a subsequent elimination of a leaving group from a tetrahedral intermediate. Both steps are enhanced by conversion of the glutamate carboxyl group into a mixed carboxylic-phosphoric anhydride. The phosphoryl group of the mixed carboxylic-phosphoric anhydride is best represented in a dipolar form, with a positively charged phosphorus and a negatively charged oxygen,^22^ which explains its electron-withdrawing effect. Thus, the phosphoryl group pulls, through an inductive effect, the electron density away from the glutamate carbonyl carbon atom, thereby making it more electrophilic and, hence, more amenable to attack by nucleophiles, such as Lys-NH_2_. Apart from this, the conversion of the initial glutamate hydroxyl group into diethyl phosphate, which is a much better leaving group, accelerates the elimination step and hence the formation of the nucleophilic substitution product, the cross-linked peptide in this case. The two mechanisms in Figure 3 produce the identical zero-length ε-lysine γ-glutamyl isopeptide bond in cross-linked proteins. Though diethyl phosphate adducts are present in the treated proteins, the cross-linking reaction removes the bound diethyl phosphate.

Figure 3A shows the activation of glutamate by CPO, followed by isopeptide cross-link formation with lysine. Diethyl phosphate is a good leaving group for nucleophilic substitution. Thus, isopeptide cross-link formation between OP-Glu and Lys is likely.

Figure 3B shows activation of lysine by CPO, followed by isopeptide cross-link formation with glutamate. Vicinal negatively charged residues aid these reactions.^17,23^

The two mechanisms in Figure 3 produce identical zero-length ε-lysine γ-glutamyl isopeptide bonds in cross-linked proteins. The cross-linking reactions in Figure 3 are not catalyzed by an enzyme. The chemically induced cross-links in Figure 3 are between lysine and glutamate. Similar cross-links are catalyzed by transglutaminase. Transglutaminase-catalyzed isopeptide cross-links are between lysine and glutamine. Mass spectrometry easily distinguishes between cross-links to glutamate and cross-links to glutamine based on the amino acid sequences of the cross-linked peptides.

**Figure 4:**
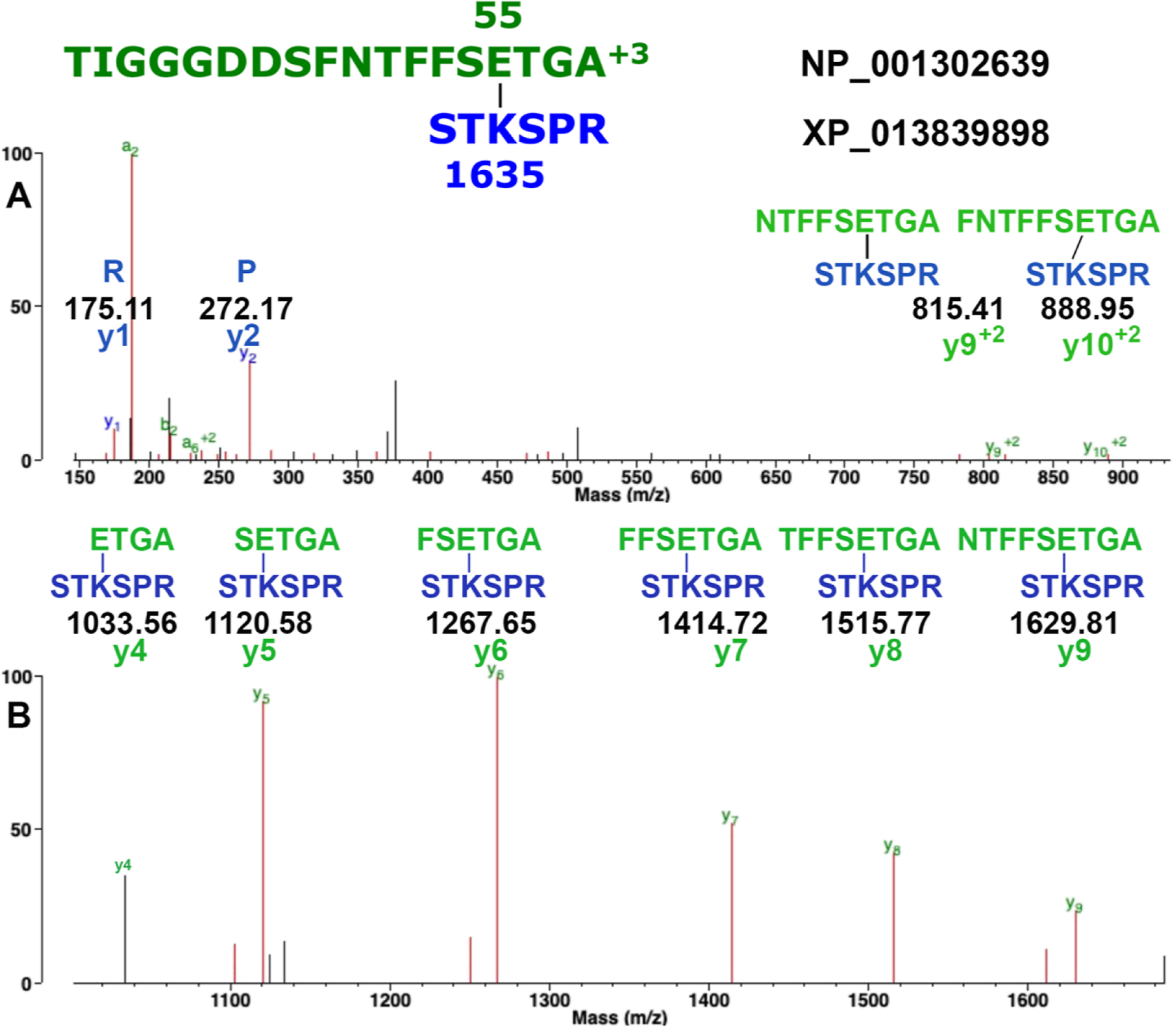
Tubulin α 1A (*S. scrofa*) is cross-linked to MAP2 X8 (*S. scrofa*) *via* an isopeptide bond between E55 of tubulin α 1A and K1635 of MAP2 X8. The cross-link was induced by incubation of MAP-rich tubulin with 200 μM CPO. The structures of two doubly charged y ion cross-link specific ions are shown in panel A. The structures of six singly-charged, cross-link specific y ions are shown in panel B. These ions support the cross-linked peptide pair. Cross-link specific ions contain masses from both the green and blue peptides. The blue y1 and y2 ions are independent support for the presence of the blue peptide. The MH^3+^ parent ion has a mass of *m*/*z* 827.05. The Protein Prospector MS/MS spectrum was expanded into panels A and B to make room for the structures. This cross-linked peptide pair could have originated from either the reaction of diethylphospho-Glu + lysine or diethylphospho-Lys + glutamate.

Only two cross-linked peptide pairs are present in data sets that contain 21 diethylphospho-glutamate and diethylphospho-lysine adducts. The relatively low number of cross-linked peptides suggests that a low percentage of activated residues undergoes a cross-linking reaction. The MS/MS spectrum in Figure 4 supports the cross-link between tubulin α 1A and MAP2 X8.

**Figure 5:**
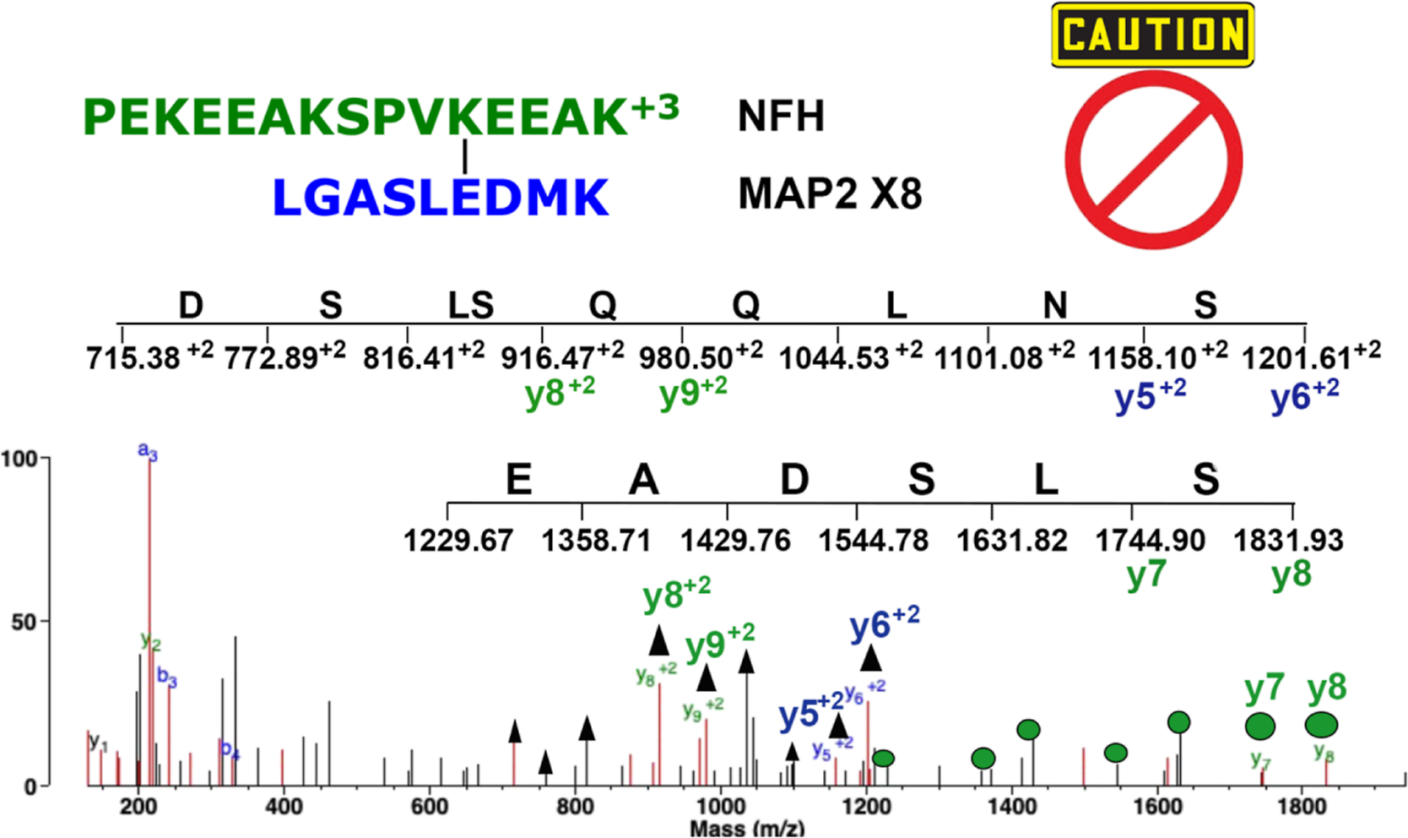
Example of a rejected candidate cross-linked peptide. This peptide pair was judged to be a false positive based on manual evaluation. The proposed cross-link specific ions, green y8^2+^, green y9^2+^, blue y5^2+^, and blue y6^2+^, were identified as part of peptide DSLSQQLNS (associated peaks indicated by the black arrows). The proposed cross-link specific ions, green y7 and green y8, are part of peptide EADSLS (associated peaks indicated by the green dots). Thus, the proposed cross-link-specific ions do not fit the candidate sequences. There is no support for either the green or blue peptide and no support for a cross-link. The MH^3+^ parent ion has a mass of *m*/*z* 881.7897.

Software searches produce thousands of candidate cross-linked peptides. A majority of them are ruled out by applying our criteria for accepting candidate cross-linked peptide pairs, including manual evaluation of MS/MS spectra. Details of our criteria for accepting candidate cross-linked peptide pairs, with an emphasis on manual evaluation, are described.^18,24^Figure 5 is an example of a candidate cross-linked peptide pair that was ruled out as a false positive. A first look at the spectrum suggested a real cross-link because there are six cross-link specific ions: green y8^2+^, green y9^2+^, blue y5^2+^, blue y6^2+^, green y7, and green y8. However, manual evaluation of the MS/MS data showed that these ions belong to peptides that have no sequence in common with the candidate cross-linked peptide pair.

### Does Diethylphospho-tyrosine Participate in Protein Cross-linking?

We are certain that CPO adds diethyl phosphate to the side chain of tyrosine in proteins. This certainty comes from use of a highly selective antidiethyl phosphate-tyrosine antibody to detect and immunopurify OP-tyrosine-modified proteins and peptides.^13^ MS/MS spectra support OP-tyrosine adducts, refer to^13^ and Figure S2. Tables 1 and 2 show a relatively high abundance of diethylphospho-tyrosine adducts. These observations suggested the possibility that a cross-linking reaction could be initiated by activated tyrosine.

**Figure 6:**
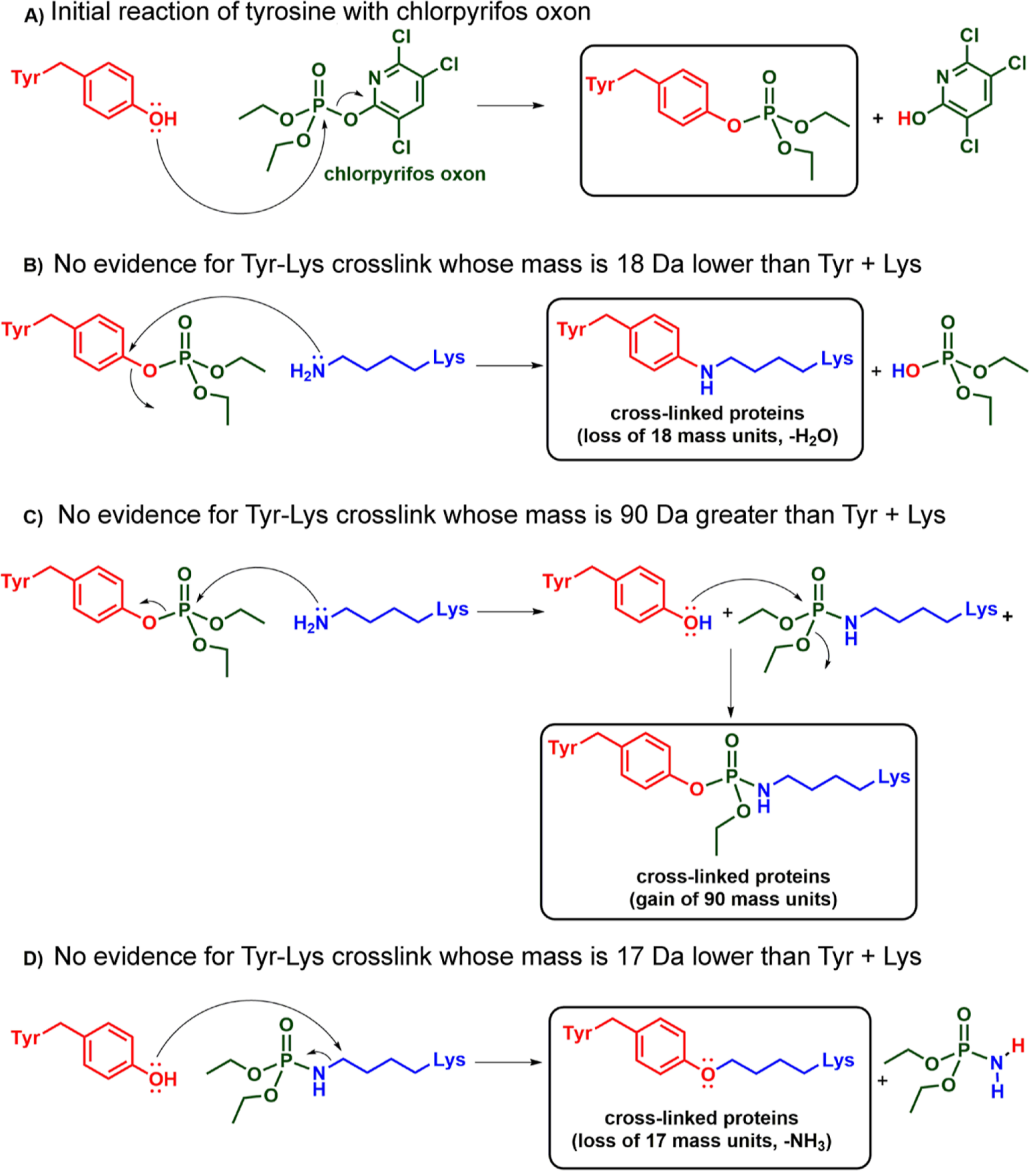
Hypothetical mechanisms for proteins cross-linked *via* a covalent bond between tyrosine and lysine. The initial reaction in A yields authentic diethoxyphospho-tyrosine. However, diethoxyphospho-tyrosine was not found to make a covalent bond with lysine.

We envisioned three mechanisms that might yield a cross-link between OP-tyrosine and lysine (see Figure 6). Mechanism B would be an aromatic nucleophilic substitution with loss of water (−18 Da). However, this reaction requires the aromatic ring to be electron deficient, which it is not. On the contrary, the aromatic ring is electron enriched by virtue of the electron-donating resonance effect of the oxygen atom. Therefore, mechanism B is not likely to occur.

Mechanism C shows a nucleophilic attack of the ε-amine from lysine on the phosphorus of OP-tyrosine. The phosphorus should be the most electro-positive atom in this construct and thus the principal site for attack by the nucleophilic amine. Because a phenoxy group is a better leaving group than an ethoxy, a phospho-lysine and free tyrosine should result. The tyrosine hydroxyl could then re-react with the phosphorus of phospho-lysine, creating an ethoxyphosphoryl containing cross-link, with an added mass of 90 Da.

Mechanism D shows an alternate reaction path between the tyrosine and OP lysine formed in the first step of mechanism C. The tyrosine hydroxyl could attack the carbon adjacent to the ε-amine in OP-lysine. This would be a standard aliphatic nucleophilic substitution on an electrophilic aliphatic carbon atom with a phosphoramidate leaving group, and as such it is likely to occur. The δ mass in this case would be −17 Da for the loss of ammonia.

We tested these hypothetical mechanisms by searching for peptides cross-linked between tyrosine and lysine with loss of 18 Da, with loss of 17 Da, and with addition of 90 Da. Our searches yielded no definitive peptide pairs linked through the side chains of tyrosine and lysine.

## Discussion

### Diethylphospho-glutamate

We have shown that CPO makes OP-lysine adducts^17^ but have not previously considered the possibility of OP-glutamate adducts. The mechanism in Figure 3A illustrates CPO reacting with glutamate to make an OP-glutamate adduct. We searched our MS files and report that OP-glutamate adducts do exist in proteins treated with CPO. This supports the possibility that cross-linking between glutamate and lysine is initiated by OP adducts on glutamate. However, OP-glutamate adducts are less abundant than OP-lysine. This could be interpreted to mean that the predominant cross-linking reactions involve OP-lysine. Alternatively, the low abundance of OP-Glu peptides could mean that they are very reactive and do not remain long after they are formed. Indeed, the electrophilic activation of the glutamate carboxyl in the mixed carboxylic-phosphoric anhydride increases the reactivity not only toward Lys-NH_2_ but also toward other adventitious nucleophiles, including water. Thus, it is known that phospho amino acids phosphoaspartate and phosphoglutamate hydrolyze readily, with their inherent instability under neutral, acidic, and alkaline conditions somewhat hampering their study in biological systems and making it necessary to use special techniques for detecting such transient species.^25^

The presence of diethylphospho-glutamate adducts generated by CPO raises the question of whether formation of glutamate-adducts can be promoted by other OP compounds. From theoretical and chemical standpoints, all OP agents should be able to create adducts on glutamate because they all contain good leaving groups. However, a retrospective examination of mass spectral data collected from MAP-tubulin samples reacted with diazoxon, paraoxon, dichlorvos, or monocrotophos found no OP adducts on glutamate.

In the current study, we have employed a rather limited set of proteins, purified MAP-tubulin and transglutaminase. However, there is no reason to believe that organophospho-glutamate adducts could not occur on other proteins, provided they have glutamate residues exposed at the surface. Consistent with this prediction, we have reported that CPO creates diethylphospho-tyrosine adducts on human albumin,^26^ human transferrin,^16^ human keratin 2, and bovine actin.^8^

Our proposed mechanisms in Figure 3 for protein cross-linking initiated by diethylphospho-glutamate and diethylphospho-lysine are supported by the following facts. (1) Diethyl phosphate adducts are present on the γ-carboxyl of glutamate and on the ε-amine of lysine in proteins treated with CPO. (2) Anti-isopeptide antibodies successfully enrich digests for isopeptide cross-linked peptides. (3) Fragment ion masses in MS/MS spectra are consistent with zero length isopeptide cross-links between glutamate and lysine. (4) Activation of carboxylic acids by phosphate reagents pulls electrons away from the carbonyl carbon, thereby increasing the electrophilicity of the carbonyl carbon^25^ for attack by the nucleophilic ε-amine of lysine. (5) There are precedents for protein cross-linking *via* an isopeptide bond: (a) the crystal structures of proteins from Gram-positive bacteria show the presence of isopeptide bonds.^23^ Isopeptide bonds provide the bacteria exceptional resistance to mechanical and thermal stress. (b) Factor XIII, a member of the transglutaminase family of enzymes, catalyzes the cross-linking of fibrin molecules *via* an isopeptide bond to form a mechanically strong blood clot.^27,28^

In our first reports on the cross-linking activity of CPO, we proposed a mechanism in which the diethyl phosphate group was on lysine.^17^ Analysis of tryptic peptides using the 6600 Triple TOF mass spectrometer identified diethyl phosphate adducts on lysine and tyrosine but on no other residues.^17^ In later trials, we analyzed our digests on the more sensitive ultrahigh-pressure liquid chromatography system coupled to an Orbitrap Fusion Lumos Tribrid mass spectrometer. Using this more sensitive system has added diethylphospho-glutamate to our list of identified diethyl phosphate adducts. This allows us to propose protein cross-linking reactions initiated by diethylphospho-glutamate and by diethylphospho-lysine.

A reviewer asked whether the nonreducible butyrylcholinesterase dimer^29^ could be an organophosphate-induced dimer. We tested this possibility by analyzing gel bands containing the nonreducible butyrylcholinesterase dimer. We found no organophosphate-induced glutamate–lysine cross-links and no transglutaminase-catalyzed glutamine–lysine cross-links in the nonreducible butyrylcholinesterase dimer. The most likely origin of the nonreducible dimer is a cross-link involving the N-linked carbohydrates of the butyrylcholinesterase tetramer. The butyrylcholinesterase tetramer is a 340 kDa sugar-coated molecule with 36 N-linked glycans^30,31^ some of which could have participated in glycation-induced protein cross-linking to yield the nonreducible dimer.^32^

Furthermore, the nonreducible dimer is also present in equine butyrylcholinesterase, human umbilical cord blood butyrylcholinesterase, and fetal bovine serum acetylcholinesterase. In contrast, recombinant human butyrylcholinesterase and recombinant human acetylcholinesterase are free of nonreducible dimers.

### Diethylphospho-tyrosine

When proteins are reacted with CPO, diethylphospho-tyrosine is the most abundant adduct formed (see Tables 1 and 2). We have presented three mechanisms by which the diethylphospho-tyrosine might lead to a cross-link with a nucleophilic amino acid such as lysine. However, we were unable to detect cross-linked peptides consistent with any of those predictions.

### Other Diethylphospho Adducts

We examined the possibility that nucleophilic amino acids other than lysine, tyrosine, or glutamate can form diethylphospho adducts upon reaction with CPO. It is firmly established that organophosphates make covalent adducts on the active site serine of serine esterases/proteases, where the catalytic triad activates one particular serine for reaction. In the current work, we found that 200 μM CPO modified Ser140 on tubulin α 1A in a reaction with purified MAP-rich tubulin. In previous work, we found FP-biotin adducts on Ser232 and Ser287 of albumin when human plasma was treated with 200 μM FP-biotin.^9^ Mice treated with intraperitoneal injections of CPO had CPO adducts on Ser338 and Tyr281 in β-tubulin isolated from mouse brain.^33^

The present work also identified a diethylphospho-adduct on threonine and on aspartate, both from human transglutaminase 2. Only arginine and histidine showed no adducts.

### Significance

Chronic low-dose exposure to OP toxicants increases the risk of impaired cognitive function and neurodegenerative diseases.^34,35^ Our findings suggest a mechanism whereby protein cross-linking is mediated by OP toxicants. High-molecular-weight cross-linked protein aggregates could disrupt cell functions, leading to illness.

## Supporting Information Available

MS/MS spectra of peptides covalently modified on lysine and tyrosine by diethyl phosphate (PDF)

## Abbreviations

MS/MS spectrum: fragmentation mass spectrum
OP: organophosphorus toxicant
CPO: chlorpyrifos oxon
SDS: sodium dodecyl sulfate
MAP: microtubule-associated protein
