# Supplementary material for: Chlorpyrifos Oxon Activates Glutamate and Lysine for Protein Cross-linking
Source: Chem Res Toxicol. 2023 Jan 4;36(1):112–21. doi: 10.1021/acs.chemrestox.2c00333 (PMC9846825; doi:10.1021/acs.chemrestox.2c00333)
Supplement: Supplementary file 1 — tx2c00333_si_001.pdf [file tx2c00333_si_001.pdf]

Chlorpyrifos oxon activates glutamate and lysine for protein crosslinking

Diego Muñoz-Torrero,† Lawrence M. Schopfer § and Oksana Lockridge §*

† Laboratory of Medicinal Chemistry (CSIC Associated Unit), Faculty of Pharmacy and Food Sciences, and Institute of Biomedicine (IBUB), University of Barcelona, , 08028 Barcelona, Spain
§ University of Nebraska Medical Center, Omaha, NE 68198 USA

E mail dmunoztorrero@ub.edu, lmschopf@unmc.edu, olockrid@unmc.edu*

Table of Contents

Page S1 Figure S1. Evidence for diethyl phosphate adduct on lysine.
Page S2 Figure S2. Evidence for diethyl phosphate adduct on tyrosine.

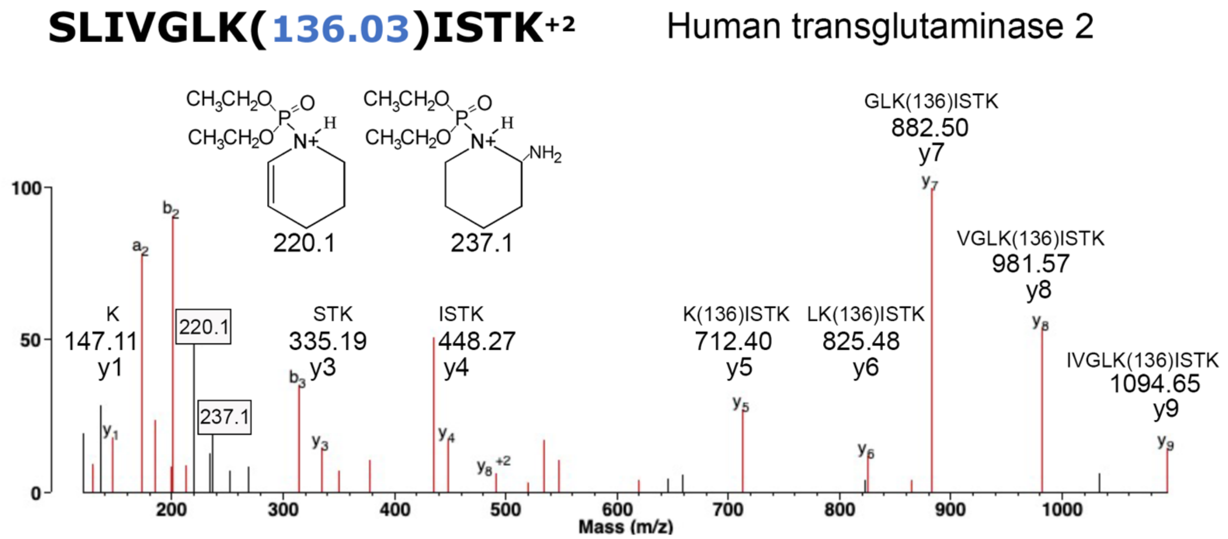

Figure S1. Evidence for diethyl phosphate adduct on lysine. The mass of the MH^{+2} parent ion of 647.8911 m/z is consistent with the peptide sequence plus 136.03 Da. The exact location of the 136.03 adduct is defined by the y4 ion at 448.27 m/z and the y5 ion at 712.40 m/z. Masses for the y1 to y4 ion series rule out the possibility that the adduct is on residues ISTK. Masses for the y5 to y9 ion series are greater by 136.03 Da than the mass of the amino acids alone, thus confirming the location of the adduct on the indicated lysine (K). The diethyl phosphate immonium ions at 220.1 and 237.1 m/z are signature ions for the diethyl phosphate adduct on lysine. Their presence supports the adduct on lysine. Human transglutaminase 2 accession number P21980.

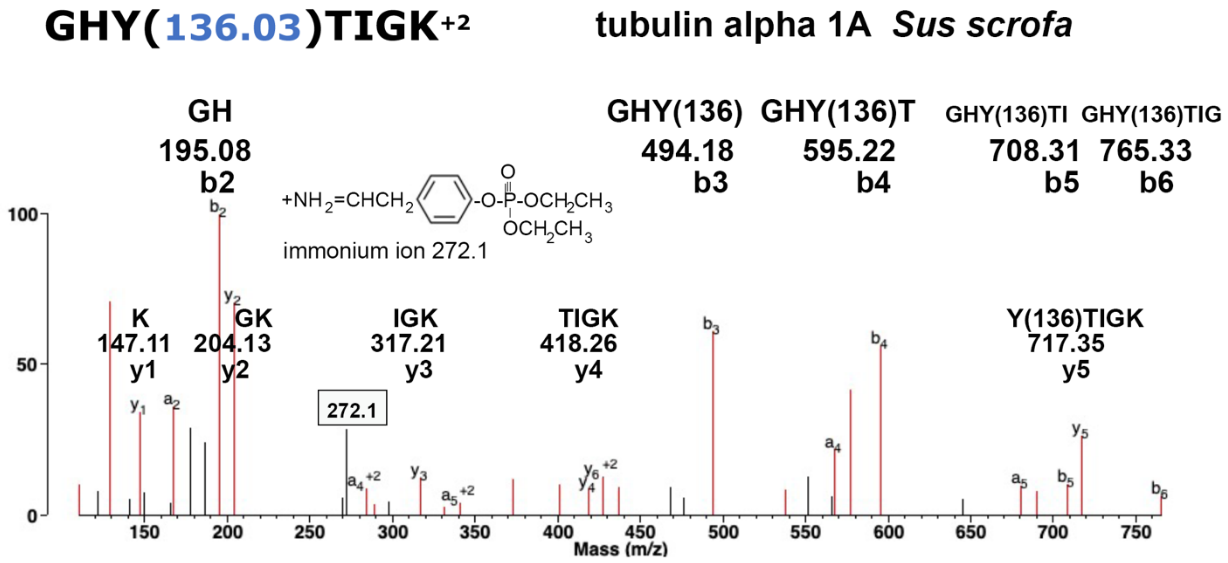

Figure S2. Evidence for diethyl phosphate adduct on tyrosine. The mass of the MH^{+2} parent ion at 456.2231 m/z is consistent with the peptide sequence plus 136.03 Da. The exact location of the 136.03 adduct is defined by the y4 ion at 418.26 m/z and the y5 ion at 717.35 m/z. Masses for the y1 to y4 ion series rule out the possibility that the adduct is on residues TIGK. The b2 to b6 ion series give strong support to tyrosine as the modified residue. The diethyl phosphate immonium ion at 272.1 m/z is the signature ion for the diethyl phosphate adduct on tyrosine. Its presence supports the adduct on tyrosine. Sus scrofa tubulin alpha 2A accession number NP_001302639.
